# A New Facility Will Harness Plasma to Guide Interplanetary Craft

**DOI:** 10.1021/acscentsci.4c01052

**Published:** 2024-07-10

**Authors:** Katherine Bourzac

On February 1, 2003, the space shuttle *Columbia* was carrying seven astronauts back to Earth after they finished work on experiments in microgravity. As the National Aeronautics and Space Administration shuttle began its reentry into Earth’s atmosphere, plasma breached the craft through a hole in its left wing, and *Columbia* broke into pieces. Everyone aboard died.

**Figure:**
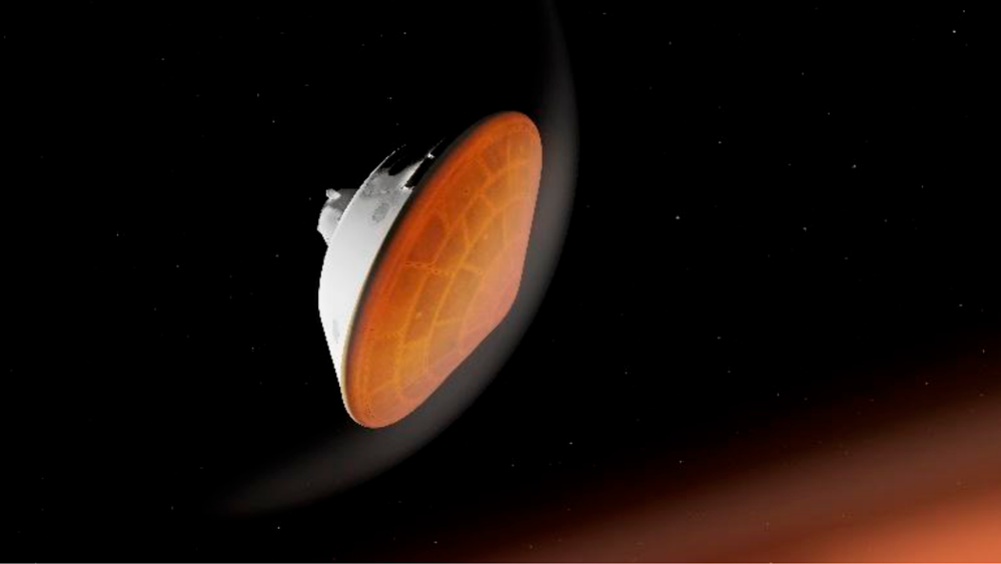
This conceptual craft would use magnets on its surface to navigate in plasma. Credit: NASA/JPL-Caltech.

Conditions for spacecraft reentering Earth’s atmosphere are dangerously intense. Vehicles traveling 5–30 times the speed of sound exert tremendous pressure on the gases surrounding them. Temperatures at a craft’s surface can surpass 10,000 K, nearly twice as hot as the surface of the sun. That’s hot enough to ionize the gases around the craft, forming a potentially destructive plasma. Aerospace engineers’ designs are constrained by the need to keep spacecraft safe from this dangerous, hot form of matter.

But since the late 1950s, aerospace engineers and physicists have been trying to figure out whether they can design spacecraft to leverage plasma rather than merely endure it. They want to build spacecraft with magnets embedded into their surfaces that can manipulate the hot, electrically charged medium in which they travel, making it possible to steer, lift, and navigate in this extreme environment. It’s an idea that could open up new vistas in aerospace engineering, making it possible to control spacecraft and military weapons, including missiles, with greater precision.

Hisham Ali, an aerospace engineer at the University of Colorado Boulder, has been studying the physics behind these proposed plasma-navigation systems since his time in graduate school, when he built a tabletop plasma wind tunnel to aid his research. Several plasma wind tunnels, which flow hot gases over test materials and parts, operate in the US. But they’re in high demand by aerospace companies, which use them to test the ability of materials and devices to stand up to intense thermal constraints. And most of these facilities aren’t ideally suited to designing and testing the science of active plasma navigation.

Now that he’s a professor, Ali is building a large-scale plasma wind tunnel at his home base in Boulder. The new tunnel will be able to make plasma that better simulates the chemistries of atmospheres on Mars and Saturn’s moon Titan, both targets of upcoming NASA missions.

**Figure:**
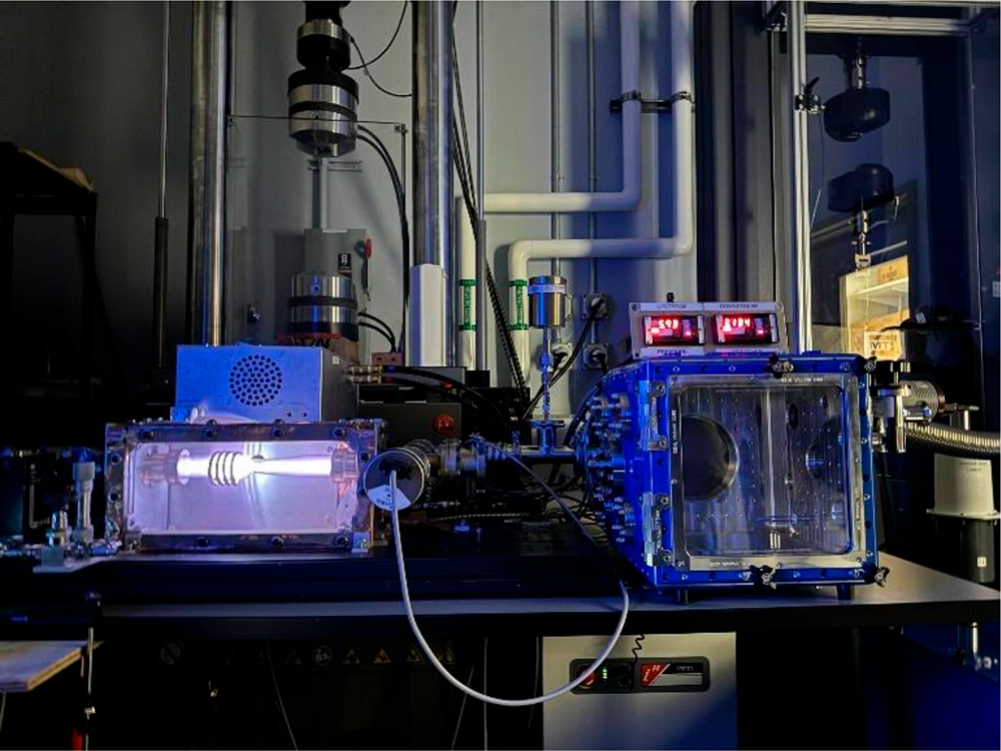
While his larger inductively coupled plasma system is being built, Hisham Ali and his students are conducting experiments in this tabletop plasma wind tunnel. Credit: Hisham Ali.

Ali started designing his plasma wind tunnel in 2022. “The whole lab has been a drawing for a long time,” he says. By this April, his team was finalizing construction.

## Plasma protection

Plasma is deadly. So it’s no wonder that aerospace engineers design for it with great care. To prevent fatal buildup of heat, engineers have a few options, says Ali.

Ballistic missiles are blunt, with no sharp edges protruding from their surfaces. This means their path can’t be easily changed during flight—any protruding rudders or ailerons would become points for deadly heat to concentrate. Most aircraft have what engineers call control surfaces that pop out at lower altitudes to help with steering but get tucked in at high altitudes when conditions are intense.

And all spacecraft are sheathed in thermal protection materials. Ali’s fascination with aeronautics starts there.

When he was in the fifth grade, he went to Space Camp in Huntsville, Alabama, and got to see part of the International Space Station before it was sent to the Kennedy Space Center for launch. “We got to touch the thermal protection tiles,” he says. “They put a blowtorch to it from the other end and let you put your hand to it.” From that moment, he knew he wanted to do work that would advance space exploration.

Ali says the inability to control the direction and speed of hypersonic craft during blazing-hot, high-velocity conditions is a major challenge when it comes to steering. “When you make trajectory corrections earlier, you have much more control over your landing site,” he says.

It’s like not being able to put your hands on the steering wheel when your car is at top speed. Significant corrections can’t be made until the craft slows down and reaches lower altitudes, when ailerons and rudders can come back out and help steer.

As things stand, Ali says, engineers can guarantee only that a craft will land within a 30 km ellipse.

Ali and others working in the field of magnetoaerodynamics hope that magnets on spacecraft can manipulate the plasma that forms on the surface of a ship and thereby steer the ship.

This idea of using plasma and magnets to help land a craft has its roots in research into the physics of how the Earth interacts with the sun, which is a giant ball of mostly plasma. The sun’s incoming ionizing radiation gets deflected by Earth’s magnetic field, keeping the hot, charged particles from incinerating us.

In 1958, two Cornell University physicists proposed that the magnetohydrodynamic effect could have aerospace applications, but this tantalizing idea has been extremely challenging to test in the lab, Ali says. Simulating the necessary conditions requires a lot of engineering finesse.

## Hot stuff

Improvements in hypersonic navigation are in great demand. Today, research in magnetoaerodynamics is driven not just by interest in interplanetary exploration but by the desire to upgrade other technologies closer to home. There’s an increasing push for hypersonic flight, defined as speeds from Mach 5 to Mach 30, for both weapons and commercial applications. At high altitudes during hypersonic flight, heat and plasma formation are also a problem.

“We’re finally getting to a point where vehicles are going so fast they get plasma generated around them,” says Mitchell Walker, an aerospace engineer at the Georgia Institute of Technology and one of Ali’s graduate thesis advisors. “Instead of just air, you have charged particles moving around.”

To study these effects, aerospace companies and world governments need plasma wind tunnels. “The demand is huge,” says Kelly Stephani, a mechanical engineer and associate director of the Center for Hypersonics and Entry Systems Studies at the University of Illinois Urbana-Champaign. The center operates a plasma wind tunnel called the Plasmatron X, which went online in 2023.

“We’re seeing growing interest in the private sector, with a lot of companies looking at various elements of hypersonic flight,” Stephani says. Plasma wind tunnels provide a place to perform ground tests of materials and parts. “There’s a bottleneck in ground-test accessibility,” she says. The US does not have enough facilities to meet the current demand, so new facilities like the Plasmatron X and the one Ali is building will offer scientists more run time and new capabilities.

Many existing facilities are of a type called arc jets. They use powerful electrodes to energize a flowing gas and form a plasma. Because the electrodes are in direct contact with the gas, impurities tend to form. Copper can sputter from the electrodes and contaminate the plasma, changing its properties. That means arc jets aren’t well suited for studying plasma when its chemical composition matters. But since they can reach blazingly high temperatures, arc jets are well suited for testing aerospace parts’ thermal properties.

But sometimes you need a pure plasma, or lower temperatures. Plasmatron X and the facility planned at UC Boulder are different from arc jets. These facilities, called inductively coupled plasma (ICP) wind tunnels, use strong radiofrequency pulses to energize an isolated gas, so the plasma never comes into contact with any electrodes. It’s contained in a tube, which is usually made of quartz, and magnetic fields constrain it so that the speeding ionized gas will flow over the object being tested without touching the container’s sides.

Ali says these systems have other advantages when it comes to simulating long flights. Typically, arc jets are limited to run times of less than an hour. The new wind tunnel will be able to run for hours, and scientists will be able to change the plasma flow rate and system power to simulate different conditions that might occur during the course of a flight.

Ali says the UC Boulder facility is only the fourth ICP wind tunnel in an academic setting in the US.

**Figure:**
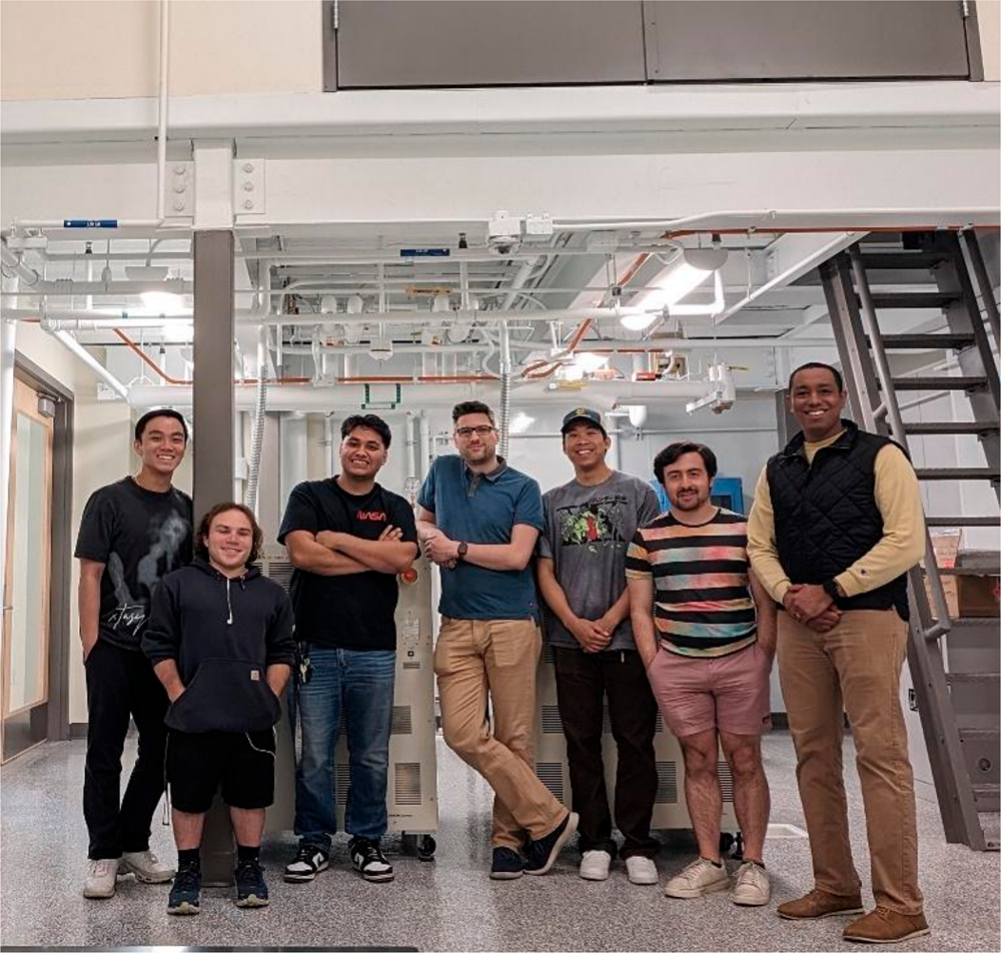
Hisham Ali (far right) and colleagues in the lab at the University of Colorado Boulder. Credit: Hisham Ali.

## Accelerating experiments

When it comes to exploring magnetoaerodynamics, chemistry is key, says Ali. Different gases create plasmas with different charge densities. And that in turn determines how the plasmas respond to magnetic fields.

To mimic Mars, scientists use canisters of carbon dioxide. To simulate conditions for NASA’s planned Dragonfly mission, which is scheduled to arrive at Saturn’s moon Titan in 2034, Ali would use nitrogen. A mix of hydrogen and helium is needed to simulate the atmospheres of our solar system’s ice giants, Neptune and Uranus. Pure plasmas provided by ICP wind tunnels are well suited for studying magnetoaerodynamic properties in situations where the chemistry matters.

ICP wind tunnels also make it possible to study materials and devices, such as communications systems, that are sensitive to electromagnetic effects. This is more challenging to implement at an arc jet. Engineers can use ICP wind tunnels to test magnetoaerodynamic devices and magnets, and to explore how hypersonic flight and plasma interfere with onboard communication systems.

Stephani says a key use of these facilities is to maintain military readiness. The US has fallen behind its adversaries in developing hypersonic weapons, Stephani says. “Now it’s catch-up time,” she says. But she says the country is progressing rapidly.

Of Ali’s project, Stephani says, “Any facility that can add additional bandwidth adds value.” Georgia Tech’s Walker agrees that Ali’s project is being built at a good time. “There’s a lot of scientific, Department of Defense, and commercial interest touching the edge of the atmosphere,” he says.

Ali expects his facility to be up and running in 2025. He’s got his mind set on a handful of first experiments. One is around the basic science of plasma. It’s still not clear how a gas’s chemistry relates to its electron-number density when ionized.

Right now, Ali is focused on simpler things. “I’d love to go and buy some of our first lab benches,” he says. And he’s thinking about what to name the facility—right now he’s contemplating “Plasmajet.”

*Katherine Bourzac is a freelance contributor to*Chemical & Engineering News*, the independent news outlet of the American Chemical Society.*

